# Achieving Net Zero—An Illustration of Carbon Emissions Reduction with A New Meta-Inverse DEA Approach

**DOI:** 10.3390/ijerph20054044

**Published:** 2023-02-24

**Authors:** Wen-Chi Yang, Wen-Min Lu

**Affiliations:** 1 Department of Diplomacy, National Chengchi University, Taipei 11605, Taiwan; 2 Department of International Business Administration, Chinese Culture University, Taipei 11114, Taiwan

**Keywords:** eco-efficiency, common but differentiated responsibilities (CBDR), carbon allocation, meta-frontier, specific super-efficiency, enhanced Russell graph measures, inverse data envelopment analysis (DEA)

## Abstract

To achieve the goal of limiting global warming to 1.5 °C above preindustrial levels, net-zero emissions targets were proposed to assist countries in planning their long-term reduction. Inverse Data Envelopment Analysis (DEA) can be used to determine optimal input and output levels without sacrificing the set environmental efficiency target. However, treating countries as having the same capability to mitigate carbon emissions without considering their different developmental stages is not only unrealistic but also inappropriate. Therefore, this study incorporates a meta-concept into inverse DEA. This study adopts a three-stage approach. In the first stage, a meta-frontier DEA method is adopted to assess and compare the eco-efficiency of developed and developing countries. In the second stage, the specific super-efficiency method is adopted to rank the efficient countries specifically focused on carbon performance. In the third stage, carbon dioxide emissions reduction targets are proposed for the developed and developing countries separately. Then, a new meta-inverse DEA method is used to allocate the emissions reduction target to the inefficient countries in each of the specific groups. In this way, we can find the optimal CO_2_ reduction amount for the inefficient countries with unchanged eco-efficiency levels. The implications of the new meta-inverse DEA method proposed in this study are twofold. The method can identify how a DMU can reduce undesirable outputs without sacrificing the set eco-efficiency target, which is especially useful in achieving net-zero emissions since this method provides a roadmap for decision-makers to understand how to allocate the emissions reduction targets to different units. In addition, this method can be applied to heterogeneous groups where they are assigned to different emissions reduction targets.

## 1. Introduction

According to the Emissions Gap Report 2021, regardless of whether countries meet their latest climate mitigation pledges, the global temperature will rise by 2.7 °C by the end of the century [1]. In his concluding remarks at the United Nations Framework Convention on Climate Change (UNFCCC) COP26 in Glasgow, UN Secretary-General António Guterres said, “Our fragile planet is hanging by a thread. We are still knocking on the door of climate catastrophe” [2]. To achieve the goal of limiting global warming to 1.5 °C above preindustrial levels, net-zero emissions targets were proposed as an important strategy to assist countries in planning their long-term reduction. The net-zero emissions strategy is also beneficial to reduce the climate change caused by extreme weather events, and its negative consequences on production systems [3,4,5,6].

With the intensification of climate change and scientists warning that humanity is running out of time, engaging all stakeholders to take climate action to reduce emissions is imperative. The 1992 UNFCCC divided the signatories into Annex I and non-Annex I countries. The Annex I countries were industrialized countries, including the Organisation for Economic Co-operation and Development (OECD) member countries and “economies in transition”. Based on the “common but differentiated responsibilities and respective capabilities” (CBDR-RC) principle adopted by the UNFCCC, the Annex I countries were called upon to “take the lead” in reducing carbon emissions, whereas no time frame was set for developing countries.

The 1997 Kyoto Protocol under the UNFCCC regime also followed the CBDR principle. The protocol considered differences in emissions, wealth, and capacity for change in the allocation of mitigation responsibilities among its signatories [7]. The Annex I countries were obligated to meet their mitigation targets, whereas legally binding mitigation targets were not imposed on the non-Annex I countries. This difference created a two-tier world [8], which was criticized by some developed countries, especially the United States, and became an impediment to climate negotiations. The Kyoto Protocol established three flexible market-based mechanisms to help the Annex I countries fulfill their obligations cost-effectively. Nevertheless, arguments about what burden-sharing scheme is appropriate as well as effective emerged. Scholars and practitioners proposed various carbon emissions allocation schemes utilizing different methods [9,10,11]. Optimization methods are based on an efficiency perspective, which can be considered a response to cost-effective advocacy. Among the optimization methods, data envelopment analysis (DEA) gained considerable popularity in environmental studies [12].

The Paris Agreement in 2015 not only expanded the participation of different countries but also resolved the dispute over only developed countries being responsible for carbon emissions reduction under the framework of the Kyoto Protocol. Instead, the countries proposed nationally determined contributions (NDC) voluntarily and determined their carbon emissions reduction contribution according to their national conditions under the Paris Agreement. Although the burden-sharing scheme seems no longer the focus of debate, how to establish mitigation plans which can effectively achieve the goal of limiting global warming to 1.5 °C persists.

Combating global warming may harm economic growth, causing many countries to hesitate in taking ambitious climate action. If countries can create more GDP with less CO_2_ emissions, in other words, more eco-efficient [13,14], then they will be more willing to commit themselves to emissions reduction. Eco-efficiency measurement via DEA can provide useful information for designing environmental strategies [15]. However, DEA was not developed for resource allocation and may undermine the effectiveness of environmental planning [16]. By contrast, inverse DEA can determine the level of inputs and outputs without sacrificing the given efficiency score. This implies that economies can reduce carbon emissions without sacrificing their economic growth.

There are at least two dimensions of the research gap. Under the framework of the Kyoto Protocol, many studies employed various DEA models to propose emissions reduction allocation schemes for countries around the world, but few adopted the inverse DEA approach. The 2015 Paris Agreement adopted a bottom-up approach instead of a top-down approach adopted by the Kyoto Protocol. After the paradigm shift, most literature employed DEA to discuss carbon quota allocation for an industry or a country, but not globally. Second, the prior literature focused on how to allocate emission reduction targets fairly, but paid less attention to how can countries utilize their resource to achieve their targets cost-effectively. Under the framework of the Paris Agreement, the countries proposed their own NDCs. It is important to set explicit short-term and long-term emission reduction targets to achieve the ultimate goal of net-zero emissions. None of the prior studies used inverse DEA to illustrate how a net-zero emission target could be achieved through resource allocation without hurting countries’ economies.

**Figure 1:**
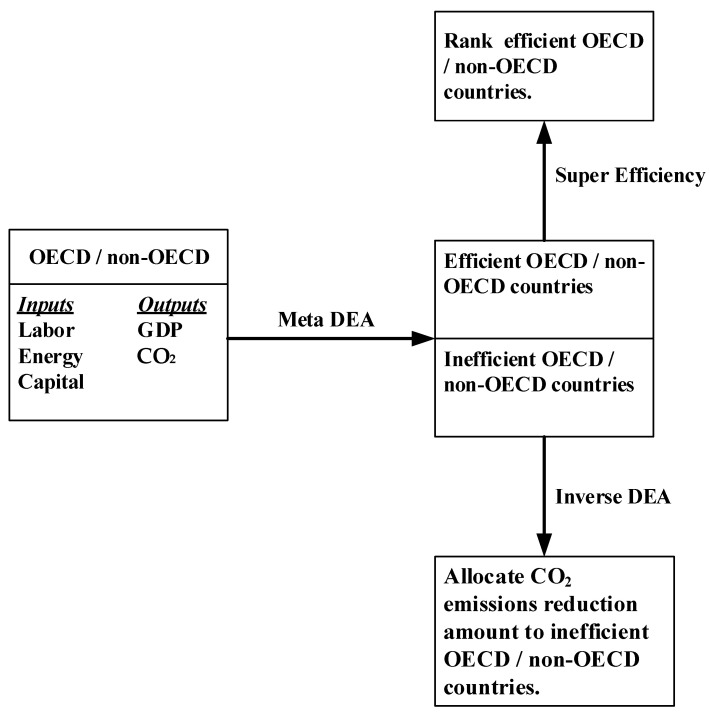
Research Framework.

To fill out the research gap, this study proposes a new meta-inverse DEA method to illustrate how can countries achieve net-zero emissions targets, and estimate the potential carbon emission reductions which can be achieved by developed and developing countries without changing their current eco-efficiency levels. Thus, this study not only extends the application to inverse DEA but also conforms to the consensus on international climate negotiations. There are three main research questions, and different approaches are employed to address each research question. First, which countries are benchmarks in terms of eco-efficiency? This study employed meta-frontier DEA to identify which countries are eco-efficient in OECD and non-OECD groups respectively. Second, how to rank the eco-efficient countries specifically focused on carbon performance? The super-efficiency was adopted for ranking. Third, how to allocate the emissions reductions target to inefficient OECD and non-OECD member countries to achieve the goal of limiting global warming to 1.5 °C? The new-meta inverse DEA is used to allocate the emissions target to OECD and non-OECD members respectively through resource planning with unchanged eco-efficiency. The research framework is illustrated in Figure 1.

The contribution of this research includes methodological refinements as well as real-world applications. Policymakers can adjust constraints based on actual situations by using this new method for achieving carbon emissions reduction targets through resource planning. In regard to methodological refinements, this research has three main contributions. First, this study integrates the meta-frontier concept into inverse DEA to enhance the method’s practical application value. Countries in different developmental stages face different social, economic, and environmental constraints as well as opportunities for production. Countries make decisions based on different sets of feasible input–output combinations constituting different technology sets. However, ignoring the differences would be misleading. Therefore, this study extends inverse DEA to meta-inverse DEA to deal with the carbon emissions reduction target allocation issue of countries worldwide. To the best of our knowledge, studies have yet to employ this method.

Second, this study replaces the radial measure with the enhanced Russell graph measure and integrates it into inverse DEA for resource management. DEA measures the relative efficiency of decision-making units (DMUs) based on two types of models. Radial models include CCR and BCC models, which were initially proposed by Charnes et al. [17] and Banker et al. [18], respectively. The efficiency scores obtained by radial models often overstate efficiency when nonzero slacks are present because they do not account for the non-radial inefficiency of the slacks. Non-radial models include additive models, multiplication models, range-adjusted measures, and slacks-based measures. The second group measures the efficiency with slacks without considering the input or output orientation. The Russell measure (RM) model belongs to the non-radial group, which was first developed by Färe and Lovell [19]. The RM model has considerable difficulty measuring efficiency because its objective function is nonlinear. Subsequently, Pastor et al. [20] proposed a modification called the “enhanced Russell graph measure”. The enhanced Russell measure can be interpreted as the ratio of the average efficiency of inputs to the average efficiency of outputs, which is more straightforward than a radial measure.

Third, this study integrates specific super-efficiency into inverse DEA to overcome the shortcomings of the tradeoff in the DEA model. Owing to the piecewise linear nature of the frontier in DEA, the estimated marginal rate of substitution applies only to infinitesimal or finitely small changes in one or more variables. The tradeoff feature of DEA determines that many of the economies with high carbon emissions in this study are located on the efficient frontier. To overcome the above problems, this study modifies the super-efficiency model and uses the specific super-efficiency model to restrict the changes in the other inputs–outputs while allowing only carbon emissions to be reduced. Therefore, this study employs specific super-efficiency to rank the efficient countries.

The rest of this paper is organized as follows. Section 2 presents the relevant literature, and Section 3 introduces the methodology. Section 4 analyzes the empirical results, and Section 5 summarizes the study and highlights some directions for further research.

## 2. Literature Review

### 2.1. Carbon Emissions Quota Allocation and DEA Application

Since the adoption of the UNFCCC in 1992, a wealth of literature initiated discussions on reduction targets or how to allocate carbon emissions quotas among countries. Various ‘effort-sharing’ or ‘burden-sharing’ approaches were proposed to achieve reduction targets. The indicator approach is the most widely adopted approach for emissions allocation, which involves the allocation of reduction targets based on a single indicator or certain indicators [21]. In the 1990s, the total carbon emissions by country became the major indicator for carbon emissions reduction based on the Kyoto Protocol [22,23]. However, the total carbon emissions of sovereign states may seem unfair to many developing countries that have just begun to experience economic growth. That is the reason why most developing countries rely on the carbon intensity indicator to fulfill their national carbon reduction targets [23]. Some researchers advocated using energy indicators for carbon emissions allocation, such as energy consumption or production because energy use is the primary source of carbon emissions [24]. Such single-factor indicators are simple to calculate and easy to understand. However, they do not reflect the actual process of CO_2_ production and neglect some important factors [25,26].

The controversial issue in the allocation of carbon emissions targets is how to establish an allocation principle that all countries will agree on and follow. Although various allocation criteria have been advocated, they may be divided into two major categories, namely equity and efficiency principles [27,28]. The equity principle can be defined from at least two aspects, that is, the “polluter pays” principle and capability [28]. The polluter pays principle was formally adopted by OECD in 1974 [10]. In the context of climate change, countries with higher emissions should assume a considerable reduction burden. Capability considers economic ability, as mitigation efforts require tremendous investments. Therefore, developed regions with high economic ability should assume a large emissions reduction burden [28]. The efficiency principle is focused on economic efficiency from the perspective of mitigation cost reduction, although some scholars argued that efficiency is also a form of fairness [29,30].

Prior studies used optimization methods with nonlinear and linear models to allocate carbon emissions targets from an efficiency perspective [31]. Filar and Gaertner [9] proposed a nonlinear model to allocate carbon emissions targets to countries globally in terms of economic utility maximization. However, the model was too complicated to use. DEA is a widely used linear programming model to allocate carbon emissions targets [31]. Gomes and Lins [32] developed a zero-sum gains DEA (ZSG-DEA) model to reallocate carbon emissions targets by putting a cap on the emissions of the industrialized and non-industrialized countries, which was subsequently employed for the carbon emissions allocation scheme in different regions. Cucchiella et al. [33] employed the above-mentioned model to allocate emissions permits among European Union member states based on their specific concerns. Färe et al. [34] proposed a DEA model to examine the magnitude and timing of carbon emissions reductions in 28 OECD countries from 1992 to 2006. DEA application was also adopted for domestic levels in several studies [34], such as Wang et al. [25] and Zhou et al. [30], which used DEA models to examine the optimal allocation of carbon emissions targets among different provinces in China. In addition, DEA models were also utilized for industrial levels to allocate carbon emissions targets. Wu et al. [35] used the DEA model for emissions quota allocation in a cap-and-trade system, whereas Sun et al. [36] adopted DEA to construct an allocation of emissions permit mechanism among a group of manufacturing companies.

Despite the wide application, Zhang et al. [37] identified defects in the use of DEA to allocate carbon emissions targets. DEA was designed for efficiency evaluation rather than resource allocation; therefore, the adjustment of resources is not the main concern. This feature may undermine the effectiveness of an allocation scheme since we emphasized the reduction of undesirable outputs. Thus, inverse DEA emerged as a practical method for studies on the allocation of carbon emissions targets.

### 2.2. Inverse DEA and Meta-Frontier DEA

DEA provides a nonparametric approach to estimating production frontiers by measuring the relative efficiency of DMUs performing similar tasks in a production system [13]. Inverse DEA deals with the reversed DEA problem and operates under the principle of inverse optimization [38]. Wei et al. [39] proposed a reverse DEA method to answer the following question: If the current efficiency level of a set of DMUs remains the same, then how much can the output increase if the inputs increase by a certain amount? In other words, inverse DEA is a type of optimization technique in the form of DEA for dealing with resource allocation problems at current efficiency levels.

Inverse DEA has been proven useful in various studies. Wei et al. [39] presented multiple objective linear programming models to estimate output levels when the DMU under assessment is inefficient [40]. Yan et al. [41] further developed inverse DEA by applying it to resource allocation. Hu et al. [42] pointed out that an inverse DEA model has two key features: its base DEA model and adopted measurement efficiency. DEA models can be categorized as radial and non-radial models. Although various inverse DEA models were developed, such as Zhang and Cui [43,44,45], they all belong under the radial model category.

Most existing inverse DEA models are based on radial DEA models, and their efficiency measure considers only radial efficiency and neglects slacks. Owing to the incomplete efficiency measure problem suffered by radial inverse DEA models, their results may be unreliable [42]. In addition, to achieve efficiency, radial inverse DEA models suggesting the reduction of resources synchronously without discriminating between different input variables may be subjective and unreasonable. In this study, we adopt the enhanced Russell graph measure, so the different variables can have different reduction ratios.

Many studies adopted DEA to analyze environmental issues under the framework of eco-efficiency or environmental efficiency. With the development and incorporation of undesirable outputs in a DEA model, research efforts explored how to improve the degree of efficiency for the operation and allocation of carbon emissions targets [46]. Traditional DEA assumes that all countries are similar without considering the heterogeneity of their capabilities to access and convert resources and reduce carbon emissions. In reality, the cost of pollution abatement and its impact on productivity tend to differ owing to variations in technology and resource availability across countries [47,48]. Using a pooling approach to measure a country’s eco-efficiency performance would be inappropriate, and contradicts the CBDR-RC principle shared in the international community [15,23]. To the best of our knowledge, no studies utilizing inverse DEA have considered heterogeneity and integrated meta-frontier approach in developing resource allocation plans.

## 3. Methodology

**Figure 2:**
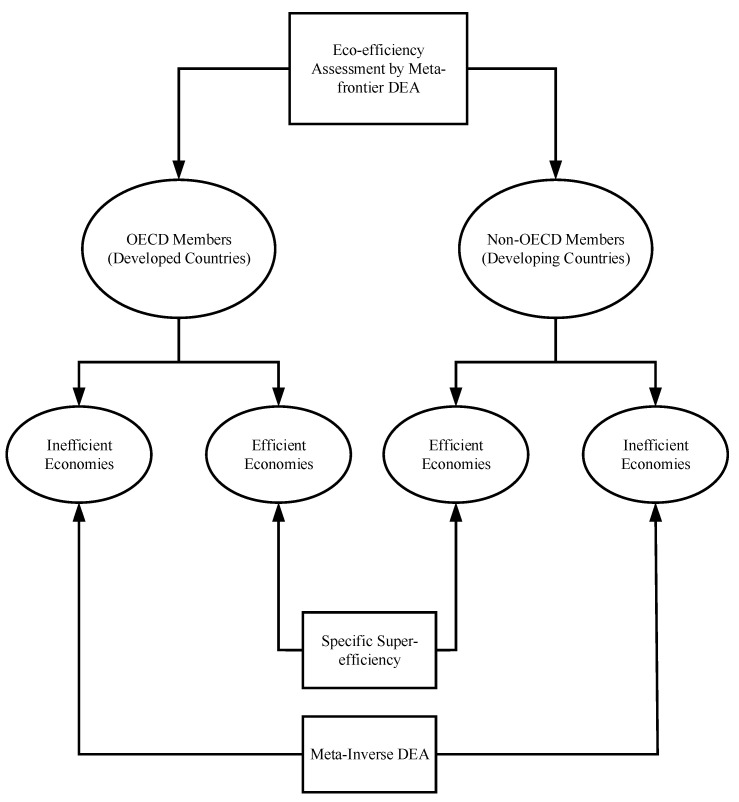
Flow Chart of the Research Methodology.

This study adopts a three-stage approach, and different methodologies are applied in different stages based on specific purposes. A research flow chart is presented in Figure 2. In the first stage, a meta-frontier DEA method is adopted to assess and compare the eco-efficiency of developed and developing countries. In the second stage, the specific super-efficiency method is adopted to rank the efficient countries specifically focused on carbon performance. In the third stage, carbon dioxide emissions reduction targets are proposed for the developed and developing countries separately. Then, a new meta-inverse DEA method is used to allocate the emissions reduction target to the inefficient countries in each of the specific groups. In this way, we can find the optimal CO_2_ reduction amount for the inefficient countries with unchanged eco-efficiency levels.

### 3.1. Meta-Frontier and Group Frontiers of the Directional Distance Function

Considering technological heterogeneity, O’Donnell et al. [49] constructed a meta-frontier and group frontiers to measure their impact on efficiency. A meta-frontier is composed of DMUs from all groups, whereas group frontiers denote individual groups. A meta-frontier is presumed to envelop all individual group frontiers. We assume a set of N DMUs that can be classified into p dissimilar groups ($G_{g},g=1,\ldots ,p$). The sample of the $G_{g}$ group is $N_{g}$, where $N_{1}+N_{2}+\ldots +N_{p}=N$. Each DMU has m inputs, s outputs, and q undesirable outputs. We use inputs $x\in R_{+}^{m}$ to produce the desired output $y\in R_{+}^{s}$ and undesirable output $b\in R_{+}^{q}$. We assume that the input and output production possibilities sets (PPS) are convex. The meta-distance directional function and two group-specific directional distance functions are

$$\begin{array}{l} {\vec{D}}^{M}\left(x,y,b;g_{x},g_{y},g_{b}\right)=Max\left\{\alpha +\beta +\zeta :\left(x-\alpha g_{x},y+\beta g_{y},b+\zeta g_{b}\right)\in T^{M}\left(x,y,b\right)\right\}.\\ {\vec{D}}^{G}\left(x,y,b;g_{x},g_{y},g_{b}\right)=Max\left\{\gamma +\tau +\rho :\left(x-\gamma g_{x},y+\tau g_{y},b+\rho g_{b}\right)\in T^{G}\left(x,y,b\right)\right\},\\ G=G_{1},\ldots ,G_{P}. \end{array}$$

We define the meta-technology and the group-specific technology sets as follows:

$T^{M}\left(x,y,b\right)$:$x\in R_{+}^{m}$ can produce desirable outputs $y\in R_{+}^{s}$ and undesirable outputs b∈ in the production process.

$T^{G}\left(x,y,b\right)$:$x\in R_{+}^{m}$ can be used by the economies in group $G_{g}$ of the first process to produce the desired outputs $y\in R_{+}^{s}$ and undesirable outputs b∈ in the production process.

In addition, the meta-technology set consists of the G group-specific technology set $T^{M}\left(x,y,b\right)=\left\{T^{G_{1}}\left(x,y,b\right)\cup \ldots \cup T^{G_{P}}\left(x,y,b\right)\right\}$. According to Fried et al. [50], the direction vector $g=\left(g_{x},g_{y},g_{b}\right)$ should be chosen before the directional distance function is evaluated. In this study, we consider the direction to be $g=\left(g_{x}=x,\;g_{y}=y,\;g_{b}=b\right)$ [51,52]. The measurement of inefficiency measure for the economies in the meta-technology and group-specific technology sets under convex constraints can be constructed as Equations (1) and (2).

(1) $$\begin{array}{l} {\vec{D}}^{M}=Max\;\sum_{i=1}^{m}\alpha_{io}^{M}+\sum_{r=1}^{s}\beta_{ro}^{M}+\sum_{h=1}^{q}\zeta_{ho}^{M}\\ S.T.\\ \sum_{g=1}^{p}\sum_{j=1}^{N_{g}}\lambda_{j}^{g}x_{ij}^{g}\leq x_{io}^{g}-\alpha_{io}^{M}g_{iox},i=1,\ldots ,m,\\ \sum_{g=1}^{p}\sum_{j=1}^{N_{g}}\lambda_{j}^{g}y_{rj}^{g}\geq y_{ro}^{g}+\beta_{ro}^{M}g_{roy},r=1,\ldots ,s,\\ \sum_{g=1}^{p}\sum_{j=1}^{N_{g}}\lambda_{j}^{g}b_{hj}^{g}\leq b_{ho}^{g}-\zeta_{ho}^{M}g_{hob},h=1,\ldots ,q,\\ \sum_{g=1}^{p}\sum_{j=1}^{N_{g}}\lambda_{j}^{g}=1,\\ \lambda_{j}^{g}\geq 0,g=1,\ldots ,p. \end{array}$$

(2) $$\begin{array}{l} {\vec{D}}^{G}=Max\;\sum_{i=1}^{m}\gamma_{io}^{g}+\sum_{r=1}^{s}\tau_{ro}^{g}+\sum_{h=1}^{q}\rho_{ho}^{g},g=1,\ldots ,P\\ S.T.\\ \sum_{j=1}^{N_{g}}\lambda_{j}^{g}x_{ij}^{g}\leq x_{io}^{g}-\gamma_{o}^{g}g_{iox},i=1,\ldots ,m,\\ \sum_{j=1}^{N_{g}}\lambda_{j}^{g}y_{rj}^{g}\geq y_{ro}^{g}+\tau_{o}^{g}g_{roy},r=1,\ldots ,s,\\ \sum_{j=1}^{N_{g}}\lambda_{j}^{g}b_{ij}^{g}\leq b_{ho}^{g}-\rho_{o}^{g}g_{hob},h=1,\ldots ,q,\\ \sum_{j=1}^{N_{g}}\lambda_{j}^{g}=1,\\ \lambda_{j}^{g}\geq 0. \end{array}$$

where $\lambda_{j}^{g}$ is the intensity variable corresponding to the processes, and $N_{1}+N_{2}+\ldots +N_{p}=N$.

Thus, operational efficiency in the meta-technology and group-specific technology sets are defined as meta-frontier technology efficiency (*MTE*) and group-specific technology efficiency (*GTE*).

$$MTE_{o}^{M}=\left[\sum_{i=1}^{m}\alpha_{io}^{M}+\sum_{r=1}^{s}\beta_{ro}^{M}+\sum_{h=1}^{q}\zeta_{ho}^{M}\right]/\left(m+s+q\right)$$

and

$$GTE_{o}^{G}=\left[\sum_{i=1}^{m}\gamma_{io}^{g}+\sum_{r=1}^{s}\tau_{ro}^{g}+\sum_{h=1}^{q}\rho_{ho}^{g}\right]/\left(m+s+q\right)$$

which is between 0 and 1. These variables indicate that the target DMU is efficient if $MTE_{o}^{M}$ and $GTE_{o}^{G}$ are equal to unity.

### 3.2. Technology Gap Ratio (TGR) and Meta-Frontier Inefficiency

As $T^{M}\left(x,y,b\right)=\left\{T^{G_{1}}\left(x,y,b\right)\cup \ldots \cup T^{G_{P}}\left(x,y,b\right)\right\}$ implies that the meta-frontier envelopes the G group-specific frontiers, the eco-efficiency measured by the meta-frontier ($MTE_{o}^{M}$) is less than that measured by the group-specific frontiers ($GTE_{o}^{G}$). Thus,

(3) $$MTE_{o}^{M}\left(x,y,b\right)\leq GTE_{o}^{G}\left(x,y,b\right)$$

The ratio of the efficiency of the meta-frontier to that of the group-specific frontiers is defined as *TGR*.

(4) $$TGR\left(x,y,b\right)=MTE_{}^{M}\left(x,y,b\right)/GTE_{}^{G}\left(x,y,b\right)\leq 1$$

The value of *TGR* is less than 1, and the closer the *TGR* to 1, the closer the group-specific frontiers are to the meta-frontier.

### 3.3. Inverse DEA Model to Allocate Carbon Emissions Targets and Super Efficiency Model

Based on Emrouznejad et al. [53], the Russell graph measure is integrated into the efficiency calculation. We perform the following procedures to allocate carbon emissions reduction targets among the designated DMUs.

First, we use Equation (5) to divide all the DMUs into two sets of efficient and inefficient DMUs, which are denoted as *F* and *L* respectively.

(5) $$\begin{array}{l} {\vec{D}}^{G}=Max\;\sum_{i=1}^{m}\gamma_{io}^{g}+\sum_{r=1}^{s}\tau_{ro}^{g}+\sum_{h=1}^{q}\rho_{ho}^{g},g=1,\ldots ,P\\ S.T.\\ \sum_{j=1}^{N_{g}}\lambda_{j}^{g}x_{ij}^{g}\leq x_{io}^{g}-\gamma_{io}^{g}g_{iox},i=1,\ldots ,m,\\ \sum_{j=1}^{N_{g}}\lambda_{j}^{g}y_{rj}^{g}\geq y_{ro}^{g}+\tau_{ro}^{g}g_{roy},r=1,\ldots ,s,\\ \sum_{j=1}^{N_{g}}\lambda_{j}^{g}b_{ij}^{g}=b_{ho}^{g}-\rho_{ho}^{g}g_{hob},h=1,\ldots ,q,\\ \sum_{j=1}^{N_{g}}\lambda_{j}^{g}=1,\\ \lambda_{j}^{g}\geq 0. \end{array}$$

*L* is composed of inefficient DMUs that must reduce undesirable outputs. We assume that the efficiency level of all the DMUs in *L* remains at least the same as that before the undesirable outputs were reduced. At the same time, we let $\varpi_{ik}^{g},\theta_{rk}^{g},\vartheta_{hk}^{g}$ be the levels of the *i*th input, *r*th desirable output, and *h*th undesirable output of the *k*th DMU, respectively. Second, we establish Equation (6) to allocate the set amount of carbon emissions targets to the different economies.

(6) $$\begin{array}{l} Min\;\sum_{i=1}^{m}\varpi_{ik}-\sum_{r=1}^{s}\theta_{rk},g=1,\ldots ,P\\ S.T.\\ \sum_{j\in F}^{}\lambda_{j}^{k,g}x_{ij}^{g}+\sum_{j\in L}^{}\varpi_{ij}\lambda_{kj}-\left(1-{\hat{\gamma }}_{ik}^{g}\right)\varpi_{ik}\leq 0,i=1,\ldots ,m,\\ \sum_{j\in F}^{N_{g}}\lambda_{j}^{k,g}y_{rj}^{g}+\sum_{j\in L}^{}\theta_{rj}\lambda_{kj}-\left(1+{\hat{\tau }}_{rk}^{g}\right)\theta_{rk}\geq 0,r=1,\ldots ,s,\\ \sum_{j\in F}^{}\lambda_{j}^{g}b_{hj}^{g}+\sum_{j\in L}^{}\vartheta_{hj}\lambda_{kj}-\left(1-{\hat{\rho }}_{hk}^{g}\right)\vartheta_{hk}^{}=0,h=1,\ldots ,q,\\ \sum_{j\in F}^{}\lambda_{j}^{k,g}+\sum_{j\in L}^{}\lambda_{kj}^{g}=1,\forall k\in L.\\ \sum_{j\in L}^{}\vartheta_{hk}^{}=Q_{h},h=1,\ldots ,q,\\ 0\leq \varpi_{ik}\leq x_{ik},\forall k\in L,i=1,\ldots ,m,\\ \left(1-C_{rk}\right)y_{rk}^{g}\leq \theta_{rk},\forall k\in L,r=1,\ldots ,s,\\ 0\leq \vartheta_{hk}^{}\leq b_{hj}^{g},\forall k\in L,h=1,\ldots ,q,\\ \lambda_{j}^{k,g}\geq 0,\forall j\in F_{k},k\in L,\\ \lambda_{kj}^{g}\geq 0,k,j\in L. \end{array}$$

Reducing undesirable outputs may cause the reduction of input and desirable output levels. The objective of Equation (6) is to minimize the total amount of inputs and maximize the desirable outputs for each DMU in *L* which needs to reduce the amount of $Q_{h}^{g}\left(h=1,\ldots ,q\right)$ from the *h*th undesirable outputs. To reflect the reality that limitations or policy thresholds for certain input or output indicators, we set constraints to limit the reduction of the desired outputs. $\left(1-C_{rk}\right)y_{rk}^{g}\leq \theta_{rk}^{g}\leq y_{rk}^{g}\left(\forall k\in L,r=1,\ldots ,s\right)$ where $C_{rk}$ is a constant given by decision-makers. In this study, $C_{rk}=0.05$, meaning that a policy of reducing the GDP by 5% at most to reduce carbon emissions is adopted. Furthermore, ${\hat{\gamma }}_{ik}^{g}$, ${\hat{\tau }}_{rk}^{g}$, and ${\hat{\rho }}_{hk}^{g}$ are parameters guaranteeing the DMU efficiency score in *L* does not decrease after the undesirable outputs are reduced, as $0\leq {\hat{\gamma }}_{ik}^{g}\leq \gamma_{ik}^{g*},0\leq {\hat{\tau }}_{rk}^{g}\leq \tau_{rk}^{g*},0\leq {\hat{\rho }}_{hk}^{g}\leq \rho_{hk}^{g*}$, where $\gamma_{ik}^{g*}$, $\tau_{rk}^{g*}$, and $\rho_{hk}^{g*}$ are the optimal values of Equation (5). Let ${\hat{\gamma }}_{ik}^{g}=\gamma_{ik}^{g*},{\hat{\tau }}_{rk}^{g}=\tau_{rk}^{g*},{\hat{\rho }}_{hk}^{g}=\rho_{hk}^{g*}$.

The inverse DEA model (6) can be simplified to the following relaxed linear programming inverse DEA model.

(7) $$\begin{array}{l} Min\;\sum_{i=1}^{m}\varpi_{ik}^{g}-\sum_{r=1}^{s}\theta_{rk}^{g},g=1,\ldots ,p\\ S.T.\\ \sum_{j\in F}^{}\lambda_{j}^{k,g}x_{ij}^{g}-\left(1-\gamma_{ik}^{g*}\right)\varpi_{ik}^{g}\leq 0,i=1,\ldots ,m,\\ \sum_{j\in F}^{N_{g}}\lambda_{j}^{k,g}y_{rj}^{g}-\left(1+\tau_{rk}^{g*}\right)\theta_{rk}^{g}\geq 0,r=1,\ldots ,s,\\ \sum_{j\in F}^{}\lambda_{j}^{g}b_{hj}^{g}-\left(1-\rho_{hk}^{g*}\right)\vartheta_{hk}^{g}=0,h=1,\ldots ,q,\\ \sum_{j\in F}^{}\lambda_{j}^{k,g}=1,\forall k\in L.\\ \sum_{j\in L}^{}\vartheta_{hk}^{g}=Q_{h}^{g},h=1,\ldots ,q,\\ 0\leq \varpi_{ik}^{g}\leq x_{ik}^{g},\forall k\in L,i=1,\ldots ,m,\\ \left(1-C_{rk}\right)y_{rk}^{g}\leq \theta_{rk},\forall k\in L,r=1,\ldots ,s,\\ 0\leq \vartheta_{hk}^{g}\leq b_{hj}^{g},\forall k\in L,h=1,\ldots ,q,\\ \lambda_{j}^{k,g}\geq 0,\forall j\in F_{k},k\in L,\\ \lambda_{kj}^{g}\geq 0,k,j\in L. \end{array}$$

*F* is composed of efficient DMUs. In most DEA models, more than one DMU is efficient, denoted by a score of 1. Likewise, in this study, more than one country shares the fully efficient status. The super-efficiency model is prominent in its capability to distinguish the “real” benchmarks among the efficient DMUs. The super-efficiency model operates as follows: the observed efficient DMU is removed from the PPS, and the distance from the observed DMU to a point located on the remaining PPS is measured. If the distance is large, then the super-efficiency of the DMU is high, because it performs much better than the other DMUs. We can get the super-efficiency score of the observed DMU by solving the following linear program.

(8) $$\begin{array}{l} Min\;\theta \\ S.T.\\ \sum_{\begin{array}{l} j\in F\\ j\neq o \end{array}}^{}\lambda_{j}^{}x_{ij}^{}\leq \theta x_{io}^{},i=1,\ldots ,m,\\ \sum_{\begin{array}{l} j\in F\\ j\neq o \end{array}}^{}\lambda_{j}^{}y_{rj}^{}\geq y_{ro}^{},r=1,\ldots ,s,\\ \sum_{\begin{array}{l} j\in F\\ j\neq o \end{array}}^{}\lambda_{j}^{}b_{ij}^{}\leq b_{ho}^{},h=1,\ldots ,q,\\ \sum_{\begin{array}{l} j\in F\\ j\neq o \end{array}}^{}\lambda_{j}^{}=1,\\ \lambda_{j}^{}\geq 0. \end{array}$$

## 4. Results and Discussions

### 4.1. Data Selection and Description

If a DMU can create considerable value with low impact, then it can be viewed as eco-efficient or environmentally efficient [9,10]. Countries transform labor, capital, and energy into a desirable output of GDP and an undesirable output of carbon emissions. If a country can generate increased GDP with few inputs and low carbon emissions, then it is more eco-efficient than others. Therefore, a DEA model can be constructed from the perspective of eco-efficiency.

To illustrate the allocation of carbon emissions reduction quotas for different countries based on eco-efficiency by the new meta-inverse DEA model, we collect the energy consumption and carbon emissions data from the US Energy Information Administration (US EIA) [54] because of its wide coverage of countries and accessibility. However, the limitation is the carbon emissions data provided by the US EIA only up to 2017. The data on actual GDP and labor are obtained from Penn World Table, version 9.1 [55]. After eliminating countries with missing data, we use the data of 149 economies around the world in 2017.

In this study, we use a meta-frontier framework to evaluate the eco-efficiency of countries for the following reasons. First, countries at different developmental stages possess different capabilities. Comparing the eco-efficiency of economies based on the assumption that they all operate under the same production boundary may lead to biased results [15,48,52]. Second, though the 2015 Paris Agreement no longer differentiated between the carbon emissions reduction obligations of developed and developing countries, it reiterated the CBDR principle. Throughout the development of the international climate governance regime, all climate conventions adhered to the CBDR principle, which emphasized its importance.

In accordance with the CBDR principle, we divided all the sample economies by the OECD membership. Among the 38 OECD member countries, only South Korea and Turkey are excluded because of insufficient data. All sample economies and their groups are illustrated in Appendix A.

Table 1 presents the descriptive statistics of all the variables of the two groups. At the same time, we perform normality tests on all the input and output variables and verify that the sample economies are not all normally distributed. A special characteristic of DEA is that it can be used on variables that are not normally distributed. Therefore, DEA is appropriate for this study.

The meta-frontier approach assumes that countries in different developmental stages operate under different production technology frontiers. To verify whether differences exist between OECD and non-OECD groups, a non-parametric rank-sum statistical analysis (Mann–Whitney U-test) is used to test the unknown distribution [56]. The results of the Mann–Whitney U-test for all the variables between the OECD and non-OECD groups are reported in Table 2. The statistical results of all the variables, except labor, are significant, thereby indicating the existence of differences between the OECD and non-OECD members, which justifies the applicability of the meta-frontier framework.

### 4.2. Meta-Frontier Efficiency Analysis

Based on the meta-frontier assumption, three eco-efficiency indices are estimated for all economies in the OECD and non-OECD groups. The meta-frontier technology efficiency (MTE) results show how each country performs compared with all the other countries, while the group-specific technology efficiency (GTE) indicates the performance of a country compared with its peers in the subgroup only. The value of MTE and GTE is between 0 and 1. The closer the score is to 1, the better the country performs from the perspective of eco-efficiency. Table 3 demonstrates that the average MTE of the OECD countries is 0.769, which is higher than that of the non-OECD countries (the score is 0.718). The results are conceivable, as developed countries possess advanced technologies as well as sophisticated managerial capabilities. The average GTE of OECD economies is 0.835, while the average GTE of non-OECD economies is 0.75. The difference between GTE and MTE of OECD countries is much bigger than that of non-OECD members. The result indicates that the eco-efficiency performance of OECD countries is much more similar. The developing countries are very diversified, including emerging markets that enjoy rapid economic growth as well as least developing countries where most of the population still suffers from extreme poverty. The result implies that different developmental stages have an actual impact on a country’s eco-efficiency.

**Figure 3:**
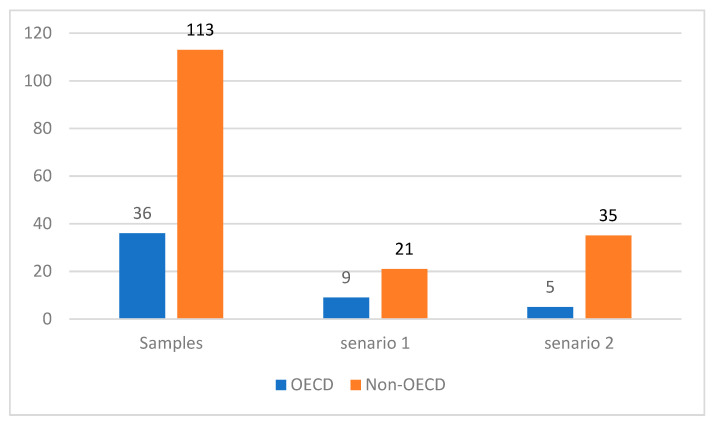
The Number of Two Scenarios for Different Groups. Note: Scenario one: GTE = MTE = 1 (the true global leaders). Scenario two: GTE = MTE.

The technology gap ratio (TGR) results, which show how close the subgroup frontier is to the meta-frontier, present a different picture. The average TGR of the OECD countries is 0.928, which is lower than that of the developing countries. In general, a TGR equal to 1 implies that an economy is located on the subgroup frontier as well as on the meta-frontier. A country achieving unity of TGR means that the country is not only the benchmark among its peers in the subgroup, but also the global leader among all countries. However, two scenarios can make the TGR equal to 1 (please refer to Figure 3). In Scenario one, a country achieves a score of 1 in its GTE as well as in its MTE, which means that the country is on the subgroup frontier as well as on the meta-frontier. In Scenario two, the GTE and MTE scores of a country are less than 1 but the same. Clearly, only the first scenario can identify the true global leader. Therefore, distinguishing between the two scenarios is necessary. Figure 3 shows the number of countries with the two scenarios in the OECD and non-OECD groups. Among the countries in the OECD group whose TGR reaches unity, 35% are global leaders. Meanwhile, among the non-OECD countries, only 18% are global leaders. The above analysis explains that the high TGR score of the non-OECD group is not because it has a higher share of global leaders but because many of its members have identical GTE and MTE scores.

### 4.3. Specific Super Efficiency Analysis

The GTE value reveals which countries are the most efficient in their subgroup. The empirical results demonstrate that many countries locate on the efficiency frontier (the GTE score is 1) in the OECD and non-OECD groups. In the OECD group, as many as 44% of the countries are located on the frontier. The empirical results raise some questions. First, can we rank all the efficient countries by order? Second, as a composite measurement of eco-efficiency, radial DEA assumes proportional improvements of inputs or outputs. It is not valid when there is a preference for improvement of a particular set of performance measurements. For instance, from the perspective of reducing GHG emissions, which country is the most efficient among its peers? For this purpose, we fix all the other input and output variables and allow only the adjustment of carbon emissions. Then, we use the super-efficiency method to rank the efficient countries based on their super-efficiency value (SEV). By doing so, we can focus only on the CO_2_ efficiency of all the sample countries.

Table 4 lists all the efficient countries in their subgroup. After adopting the specific super-efficiency method, we can clearly identify the new ranking of all the efficient countries. According to their SEV, the top emitters in the non-OECD group, namely, China, India, Brazil, and Indonesia, demonstrate an evident decrease in their efficiency scores. The second-largest emitter, namely, the United States, ranks 13 among the 16 efficient countries in the OECD group with respect to the SEV.

### 4.4. Inver DEA Results and the Allocation of CO_2_ Emissions Reduction

The first stage of inverse DEA is to distinguish which countries are inefficient, and the second stage is to allocate the carbon emissions reduction targets to the inefficient countries. Based on Equation (7), the total carbon emissions reduction targets are allocated to the inefficient countries in OECD and non-OECD groups.

The Emissions Gap Report 2021 [1] suggested that to meet the Paris Agreement, the world must reduce carbon emissions by 30% to limit global warming to 2 °C and by 55% to limit global warming to 1.5 °C. Stocker [57] concludes that “economic models estimate that feasible maximum rates of emissions reduction may not exceed about 5% per year”. Thus, we set the emissions reduction targets as 5% of the sample countries’ total carbon emissions each year. From the previous discussion, we determine that requiring developing countries to assume the same mitigation burden as developed countries would be unrealistic and unfair. Although there are various carbon reduction allocation principles such as [57,58], this study focused on how inefficient countries can achieve the reduction targets collectively. As an illustration, we assign 70% of the reduction target to the OECD countries and 30% to the non-OECD countries based on the perspective of historic responsibility and different capabilities which constitutes the cornerstone of CBDR [59,60,61]. Then, we perform the new meta-inverse DEA to allocate the reduction targets among the developed and developing countries. The process is explained in Appendix B. The results are presented in Table 5 and Table 6.

The average carbon emissions of the 21 inefficient OECD countries are 124.49 million tons, among which Canada, Australia, and Japan are the largest emitters, with emissions of 624, 411, and 300 million tons, respectively. The inverse DEA results suggest that Spain should reduce its carbon emissions the most, followed by Canada and Australia. Meanwhile, Lithuania and Latvia hardly need to reduce their carbon emissions.

For the inefficient non-OECD group, South Africa, Thailand, and Taiwan have the highest carbon emission. The carbon emission of South Africa is 10 times higher than the average emission amount in this group. Thailand and Taiwan are 7 times more than the average emissions. The inverse DEA result suggests Pakistan, Taiwan, and Thailand need to reduce the most CO_2_ emissions. Pakistan needs to cut its emissions by 25%, while Taiwan and Thailand need to cut their emissions by about 10%.

## 5. Conclusions

As climate change intensifies, engaging all stakeholders to take ambitious climate action to combat global warming is imperative. Many countries are reluctant to take ambitious climate action owing to concerns that mitigation measures may hurt their economic growth. Inverse DEA can be used to determine optimal input and output levels without sacrificing the current eco-efficiency level. Thus, the method can help decision-makers establish carbon emissions reduction strategies effectively. However, treating countries as having the same capability to mitigate carbon emissions without considering their different developmental stages is not only unrealistic but also inappropriate. This study contributes to the methodology by incorporating a meta-concept into inverse DEA. Thus, this study not only extends the application to inverse DEA but also conforms to the consensus on international climate negotiations. Based on the CBDR principle under the UNFCCC regime, this study proposes a new meta-inverse DEA model and suggests carbon emissions reduction targets for developed and developing countries.

This study adopts a three-stage approach. The first stage uses a meta-frontier DEA method to assess and compare the eco-efficiency of developed and developing countries. The empirical results show that the OECD economies have higher average efficiency scores among all the sample economies and their individual subgroup according to their MTE and GTE. Presumably, the OECD group should also lead the non-OECD group in terms of TGR, while the results demonstrate that the non-OECD group has a higher average TGR value than that of the OECD group. By further investigation, the authors find that it is necessary to clarify two different situations to identify the real global leaders who achieve unity both in MTE and GTE. Prior studies have never emphasized the difference, which may lead to a wrong conclusion.

According to Beusch et al. [62], the increase in GHGs throughout the 1991–2030 period is mostly caused by the top five emitters, namely China, the United States, the European Union (EU-27), India, and Russia. From the traditional single-factor point of view, the biggest emitters should reduce the most carbon emissions. However, from the eco-efficient point of view, the empirical results show that the largest emitters such as the United States, United Kingdom, Germany, China, and India, are efficient in their subgroup. Stabilized economic development and environmental protection are both important for human development. Eco-efficiency evaluation which is based on a cost-effective approach thus contributes to striking the balance between economic growth and environmental protection. This does not mean the big emitters can shy away from their mitigation responsibilities. Therefore, in the second stage, this study further employs the specific super-efficiency method to rank the efficient countries specifically focused on carbon emissions performance. The efficiency scores of the big emitters decreased after adopting the specific super-efficiency approach which offers the opportunity to adjust resource allocations.

In the third stage, carbon dioxide emissions reduction targets are proposed to the developed and developing countries separately based on the perspective of historical responsibility which is the cornerstone of the CBDR principle. Then, a new meta-inverse DEA method is used to allocate the emissions reduction target to the inefficient countries in each of the specific groups. In this way, we can find the optimal CO_2_ reduction amount for the inefficient countries without decreasing their current eco-efficiency level.

This research attempts to provide a new method for achieving carbon emissions targets through resource planning. Policymakers can adjust constraints based on actual situations. The implications of the new meta-inverse DEA method proposed in this study are twofold. The method can identify how a DMU can reduce undesirable outputs without sacrificing the set eco-efficiency target, which is especially useful in achieving a net-zero target since this method provides a roadmap for decision-makers to understand how to allocate the emissions reduction targets to different units. In addition, this method can be applied to heterogeneous groups where they are assigned to different emissions reduction targets.

There are some limitations of this study.

- Due to data availability, this study uses the 2017 data to illustrate the applicability of the new meta-inverse DEA. If complete data sets are available, the sample countries and study period can be expanded.
- This study is lacking a time-series analysis. Future studies can try to integrate the meta frontier Malmquist performance index (MMPI) proposed by [63] to inverse DEA.

This study shows that this method can be operationalized. Future studies can employ this method to solve resource allocation problems across a wide range of disciplines. This method can also be applied to different levels, such as regional or firm levels. Although the large emitters discussed in this study are efficient from the perspective of environmental efficiency, they should not shy away from their responsibility of reducing carbon emissions. Future research can compare suggested carbon emissions reduction targets based on different allocation principles. In addition, future studies can further examine factors that can affect environmental efficiency.

## Figures and Tables

**Table 1:** Descriptive Statistics of all Input and Output Variables of Two Groups.

Group	Variable	Unit	Mean	Median	Min	Max	Variance	SD	Normality Test
OECD	Labor	Million people	16.03	4.80	0.19	154.44	821.00	28.66	<0.01 ***
	Energy	Quad Btu	6.23	1.50	0.09	97.60	268.00	16.36	<0.01 ***
	Capital	Billion US dollars	5320.72	1866.21	52.67	56,215.31	103,078,497.00	10,152.76	<0.01 ***
	GDP	Billion US dollars	1342.58	390.02	15.33	17,711.02	9,178,997.00	3029.69	<0.01 ***
	CO_2_	Million metric tons	330.48	70.64	3.58	5133.44	751,128.00	866.68	<0.01 ***
Non-OECD	Labor	Million people	21.39	3.72	0.05	791.69	8088.00	89.93	<0.01 ***
	Energy	Quad Btu	2.54	0.22	0.00	139.43	179.00	13.39	<0.01 ***
	Capital	Billion US dollars	2003.34	184.91	3.35	94,903.73	89,362,528.00	9453.18	<0.01 ***
	GDP	Billion US dollars	493.01	50.80	0.79	18,978.50	3,938,664.00	1984.61	<0.01 ***
	CO_2_	Million metric tons	176.28	9.49	0.16	10,486.98	1,008,702.00	1004.34	<0.01 ***

Note: The asterisks *** indicate significance level of 1%.

**Table 2:** Mann–Whitney U-test of all variables.

	Rank-Sum of OECD Group	Rank-Sum of Non-OECD Group	U	Z	*p*-Level
Labor	3079	8246	1691	1.589	0.112
Energy	3854	7471	916	4.999	<0.01 ***
Capital	3949	7376	821	5.417	<0.01 ***
GDP	3838	7487	932	4.929	<0.01 ***
CO_2_	3756	7.569	1014	4.568	<0.01 ***

Note: The asterisks *** indicate significance level of 1%.

**Table 3:** Descriptive Statistics of MTE, GTE, and TGR of Two Groups.

		Valid N	Mean	Min	Max	SD
OECD	MTE	36	0.769	0.508	1.000	0.170
	GTE	36	0.835	0.514	1.000	0.171
	TGR	36	0.928	0.558	1.000	0.113
Non-OECD	MTE	113	0.718	0.390	1.000	0.176
	GTE	113	0.750	0.390	1.000	0.172
	TGR	113	0.957	0.711	1.000	0.065
All Economies	MTE	149	0.730	0.390	1.000	0.175
	GTE	149	0.771	0.390	1.000	0.175
	TGR	149	0.950	0.558	1.000	0.080

**Table 4:** Specific Super Efficiency Value of Efficient Countries.

Group	Country	SEV	Group	Country	SEV
OECD	Ireland	1.467	Non-OECD	Sao Tome and Principe	1.811
	Switzerland	1.392		Nigeria	1.756
	Poland	1.201		Sri Lanka	1.578
	Costa Rica	1.172		Iraq	1.265
	United Kingdom	1.157		Gabon	1.219
	Luxembourg	1.110		Equatorial Guinea	1.159
	Germany	1.085		Mali	1.138
	Norway	1.030		Grenada	1.045
	France	0.982		Qatar	1.005
	Estonia	0.976		Kuwait	1.000
	Mexico	0.945		Azerbaijan	0.987
	Japan	0.930		United Arab Emirates	0.952
	United States	0.809		Saudi Arabia	0.952
	Colombia	0.705		Chad	0.929
	Italy	0.643		India	0.928
	Iceland	0.546		Egypt	0.905
				Brazil	0.855
				Seychelles	0.840
				China	0.709
				Uganda	0.651
				Indonesia	0.635
				Ethiopia	0.548
				Aruba	0.456
				Bhutan	0.427

**Table 5:** Actual and Targeted CO_2_ Emissions for Inefficient OECD Countries.

Country	Reduction Amount	Actual Emissions	Country	Reduction Amount	Actual Emissions
Australia	188.30	411.10	Israel	28.24	72.20
Austria	46.89	69.07	Latvia	0.00	8.09
Belgium	43.22	137.84	Lithuania	0.00	14.77
Canada	230.63	624.83	Netherlands	76.50	240.94
Chile	48.59	87.26	New Zealand	34.17	40.09
Czech Republic	31.33	110.78	Portugal	27.71	60.21
Denmark	35.46	36.20	Slovakia	15.67	35.63
Finland	25.00	45.50	Slovenia	6.53	13.90
Greece	23.82	75.84	Spain	258.17	300.39
Hungary	27.97	52.46	Sweden	51.56	52.64

**Table 6:** Actual and Targeted CO_2_ Emissions for Inefficient non-OECD Countries.

Country	Reduction Amount	Actual Emissions	Country	Reduction Amount	Actual Emissions
Albania	1.347	4.555	Malawi	0.438	1.027
Algeria	25.386	142.315	Malaysia	26.348	228.217
Angola	9.595	19.380	Maldives	0.239	1.767
Argentina	25.217	210.133	Malta	0.591	8.849
Armenia	1.896	5.809	Mauritania	0.452	2.616
Bahrain	1.338	40.268	Mauritius	0.653	6.650
Bangladesh	20.837	82.120	Mongolia	0.801	19.748
Barbados	0.095	1.709	Montenegro	0.495	2.373
Belarus	5.045	60.790	Morocco	8.115	55.710
Belize	0.000	0.580	Mozambique	0.618	10.036
Benin	0.464	6.621	Namibia	0.740	3.982
Bosnia	0.826	18.705	Nepal	2.510	7.681
Botswana	0.936	7.676	Nicaragua	1.125	5.265
Bulgaria	3.513	45.158	Niger	0.334	2.441
Burkina Faso	0.606	3.297	North Macedonia	0.679	6.602
Burundi	0.000	0.222	Oman	5.505	67.007
Cabo Verde	0.123	0.866	Pakistan	49.921	197.059
Cambodia	1.184	10.742	Panama	2.669	26.988
Cameroon	2.699	7.074	Paraguay	2.291	8.763
Central African	0.125	0.381	Peru	12.490	53.356
Comoros	0.000	0.277	Philippines	33.389	129.872
Croatia	4.748	17.281	Romania	25.353	75.590
Cyprus	0.632	8.115	Rwanda	0.457	0.923
Djibouti	0.115	0.862	Saint Lucia	0.000	0.469
Dominican Republic	5.679	22.017	Senegal	1.407	9.337
Ecuador	4.521	37.237	Serbia	2.350	47.086
El Salvador	1.720	6.351	Sierra Leone	0.234	0.920
Eswatini	0.535	1.092	Singapore	11.511	256.523
Fiji	0.178	2.456	South Africa	14.718	476.866
Georgia	2.313	9.907	Sudan	7.296	20.178
Ghana	7.353	15.001	Suriname	0.152	2.095
Guatemala	4.005	17.985	Taiwan	37.519	324.679
Guinea	0.693	2.746	Tajikistan	1.176	6.093
Guinea-Bissau	0.000	0.311	Thailand	34.745	342.800
Haiti	0.346	3.420	Togo	0.211	2.755
Honduras	1.140	8.767	Trinidad	0.826	50.313
Jamaica	0.414	8.852	Tunisia	3.412	24.894
Jordan	2.646	27.187	Turkmenistan	2.837	99.518
Kazakhstan	9.753	295.522	Ukraine	9.491	204.483
Kenya	5.121	18.022	Uruguay	3.606	7.283
Kyrgyzstan	1.025	8.460	Uzbekistan	9.887	95.714
Lebanon	2.294	27.190	Yemen	1.524	9.649
Lesotho	0.180	0.675	Zambia	2.491	5.032
Liberia	0.087	1.201	Zimbabwe	0.576	9.976
Madagascar	0.993	3.498			

## Data Availability

All data will be available on reasonable request.

## Appendix A

**Table A1:** Classification of Sample Economies.

Group	Economy	N
OECD	Austria *, Australia *, Belgium *, Canada *, Chile, Colombia, Costa Rica, Czech Republic *, Denmark *, Estonia *, Finland *, France *, Germany *, Greece *, Hungary *, Iceland *, Ireland *, Israel, Italy *, Japan *, Latvia *, Lithuania *, Luxembourg *, Mexico, Netherlands *, New Zealand *, Norway *, Poland *, Portugal *, Slovakia *, Slovenia *, Spain *, Sweden *, Switzerland *, United Kingdom *, United States *	36
Non-OECD	Albania, Algeria, Angola, Argentina, Armenia, Aruba, Azerbaijan, Bahrain, Bangladesh, Barbados, Belarus *, Belize, Benin, Bhutan, Bosnia and Herzegovina, Botswana, Brazil, Bulgaria *, Burkina Faso, Burundi, Cabo Verde, Cambodia, Cameroon, Central African Republic, Chad, China, Comoros, Croatia, Cyprus, Djibouti, Dominican Republic, Ecuador, Egypt, El Salvador, Equatorial Guinea, Eswatini, Ethiopia, Fiji, Gabon, Georgia, Ghana, Grenada, Guatemala, Guinea, Guinea-Bissau, Haiti, Honduras, India, Indonesia, Iraq, Jamaica, Jordan, Kazakhstan, Kenya, Kuwait, Kyrgyzstan, Lebanon, Lesotho, Liberia, Madagascar, Malawi, Malaysia, Maldives, Mali, Malta, Mauritania, Mauritius, Mongolia, Montenegro, Morocco, Mozambique, Namibia, Nepal, Nicaragua, Niger, Nigeria, North Macedonia, Oman, Pakistan, Panama, Paraguay, Peru, Philippines, Qatar, Romania, Rwanda, Saint Lucia, Sao Tome and Principe, Saudi Arabia, Senegal, Serbia, Seychelles, Sierra Leone, Singapore, South Africa, Sri Lanka, Sudan, Suriname, Taiwan, Tajikistan, Thailand, Togo, Trinidad and Tobago, Tunisia, Turkmenistan, Uganda, Ukraine, United Arab Emirates, Uruguay, Uzbekistan, Yemen, Zambia, Zimbabwe	113

Note: * indicates the Annex I countries to UNFCCC.

## Appendix B

This study first set a CO_2_ emissions reduction target to limit global warming to 1.5 °C. Then applying the new meta-inverse DEA we proposed to allocate the amount to the developed and developing countries respectively based on the CBDR principle. As an illustration, 70% of the reduction target is assigned to developed countries and 30% to developing countries. Then Equation (7) is employed to calculate the reduction amount for each inefficient country with unchanged efficiency.

**Table A2:** The Process for Allocating Reduction Targets Using New Meta-Inverse DEA.

Steps		Descriptions	Amount
Step 1. Determine the CO_2_ emissions target.		5% of the total CO_2_ emissions of the sample countries	33,593.3 × 0.05 = 1679.665
Step 2. Allocate the emissions targets to the inefficient countries in the two groups.	Developed countries	70% of the CO_2_ emissions target	1679.665 × 0.7 = 1199.76
	Developing countries	30% of the CO_2_ emissions target	1679.665 × 0.3 = 479.905
Step 3. Use the new meta-inverse DEA method for calculation.	Developed countries	Calculate the CO_2_ reduction amount of each inefficient country in OECD group using Equation (7) to meet the 70% reduction target collectively.	Please refer to Table 5 for results.
	Developing countries	Calculate the CO_2_ reduction amount of each inefficient country in the non-OECD group using Equation (7) to meet the 70% reduction target collectively.	Please refer to Table 6 for results.
